# Two Hypotheses About Climate Change and Species Distributions

**DOI:** 10.1111/ele.70134

**Published:** 2025-05-08

**Authors:** John M. Drake, John P. Wares, James E. Byers, Jill T. Anderson

**Affiliations:** ^1^ Odum School of Ecology University of Georgia Athens Georgia USA; ^2^ Center for the Ecology of Infectious Diseases University of Georgia Athens Georgia USA; ^3^ Department of Genetics University of Georgia Athens Georgia USA

**Keywords:** climate change, global change, macroecology, species distribution

## Abstract

Species' distributions are changing around the planet as a result of global climate change. Most research has focused on shifts in mean climate conditions, leaving the effects of increased environmental variability comparatively underexplored. This paper proposes two new macroecological hypotheses—the *variability damping hypothesis* and the *variability adaptation hypothesis*—to understand how ecological dynamics and evolutionary history could influence biogeographic patterns being forced by contemporary large‐scale climate change across all major ecosystems. The variability damping hypothesis predicts that distributions of species living in deep water environments will be least affected by increasing climate‐driven temperature variability compared with species in nearshore, intertidal and terrestrial environments. The variability adaptation hypothesis predicts the opposite. Where available, we discuss how the existing evidence aligns with these hypotheses and propose ways in which they may be empirically tested.

## Graphical abstract

Species' distributions are changing around the planet as a result of global climate change. Most research has focused on mean climate conditions, with the effects of increased environmental variability comparatively underexplored. This paper proposes two new macroecological hypotheses—the variability damping hypothesis and the variability adaptation hypothesis—to understand how ecological dynamics and evolutionary history may influence biogeographic patterns being forced by contemporary large‐scale climate change.

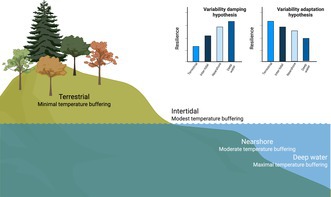

Global change has shifted species' distributions to poleward latitudes and upslope elevations on land and greater depths at sea (Lawlor et al. 2024; Lenoir et al. 2020; Lenoir and Svenning 2015; Pinsky et al. 2019; Rubenstein et al. 2023), altered the timing of critical life history events (Cooper et al. 2019), increased the risk of extinction for native species (Cahill et al. 2013), and affected the spread of pathogens (Singh et al. 2023). Investigating the proximate factors that limit contemporary geographic ranges could allow us to make more robust predictions about future distributions. While most research has focused on shifts in mean climate conditions and predictable periodic patterns (e.g., seasonality), the prospect of increased environmental variability has been comparatively underexplored (Drake 2005; Morley et al. 2017; Slein et al. 2023; Vázquez et al. 2017) despite appeals for experimentalists to manipulate variability (Miner and Vonesh 2004; Thompson et al. 2013). Furthermore, extreme events, such as heatwaves, are projected to increase in frequency and severity under climate change, posing serious threats to biodiversity (Masson‐Delmotte et al. 2023; Murali et al. 2023). Intensifying heatwaves reflect a broader trend of increasing temporal variability in global temperatures, which is projected to continue and surpass historical bounds (Masson‐Delmotte et al. 2023; Till et al. 2019). Increasing climatic variability can alter species' distributions in unexpected ways that cannot be predicted from changes in average conditions (Marshall et al. 2021; Thompson et al. 2013). However, species adapted to fluctuating or heterogeneous environments may exhibit plasticity or adaptations that increase fitness in marginal environments (Chevin and Lande 2011) or under more variable conditions associated with climate change.

Here, we seek to unite the interest of population ecologists in environmental variability and the biogeographical focus on macroecological patterns. We propose two new macroecological hypotheses aimed at deepening our understanding of how these evolutionary dynamics may influence large‐scale patterns from climate change. First, the *variability damping hypothesis* proposes that species inhabiting ecosystems that are more buffered against fluctuations in heat input will be less exposed to these fluctuations and thus at reduced risk of local extinction owing to climate change. Alternatively, we propose the *variability adaptation hypothesis* that species from ecosystems with high levels of temporal variation in climatic conditions will have evolved tolerance to variability, which could also enhance persistence in novel climates (Figure 1). Of course, these hypotheses are not mutually exclusive and there is also a null hypothesis, which is that baseline climate variability does not matter for responses to climate change.

**FIGURE 1:**
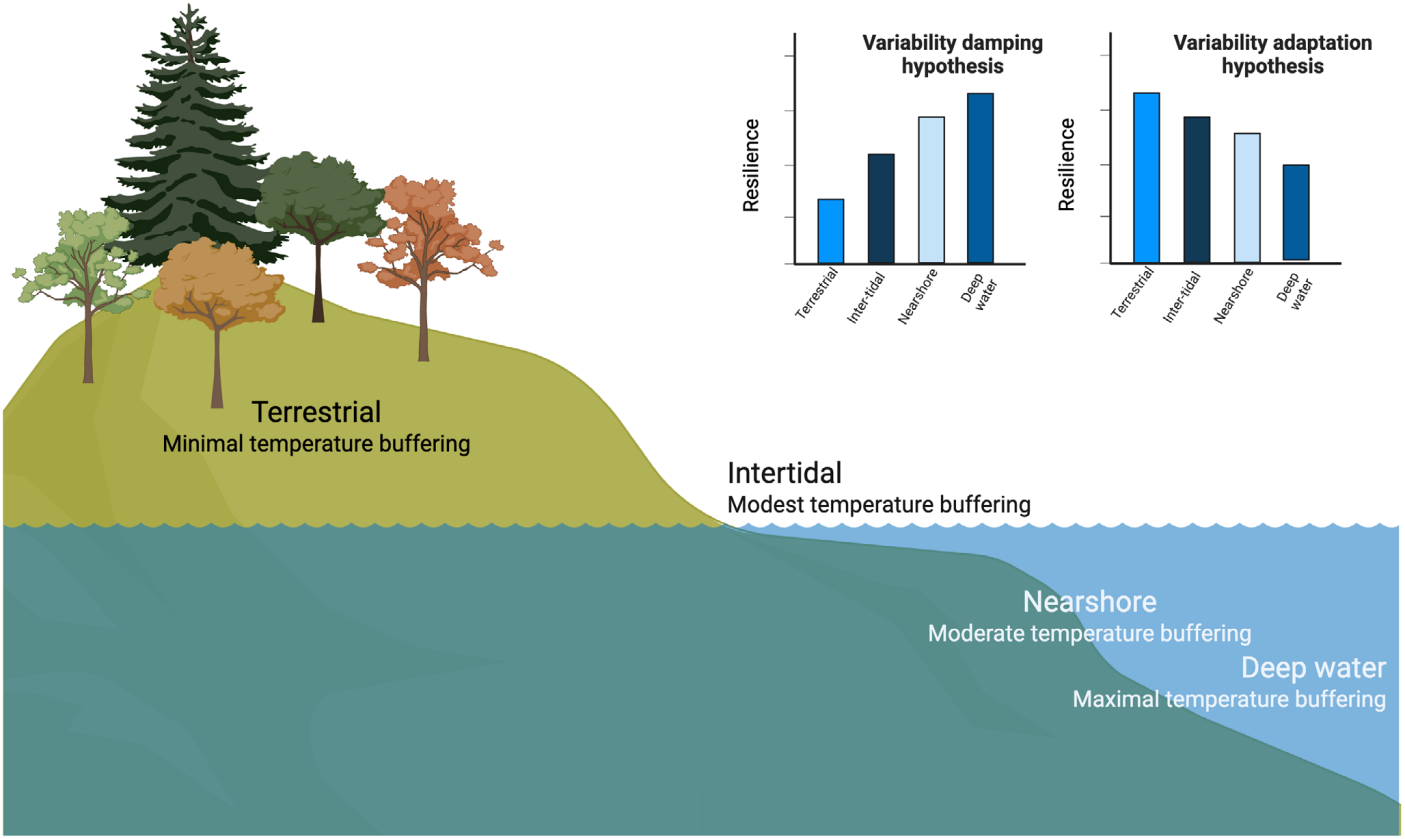
Species in different ecosystems are predicted to respond to anthropogenic climate change in different ways due to the temperature buffering capacity of their environments. Two hypotheses—the *variability damping hypothesis* and the *variability adaptation hypothesis*—make opposite predictions about the relative resilience of species in these ecosystems based on the expected magnitude of climate‐driven stressors and adaptation to historical environmental regimes. Created in BioRender. Drake, J. (2025) https://BioRender.com/q41u264.

Ecologists have been discussing the individual, population, and community impacts of increasing temporal environmental variability for two decades (Boyce et al. 2006; Drake 2005; Lawson et al. 2015; Terry et al. 2022; Vázquez et al. 2017), but so far there has been little consideration of the macroecological consequences. For instance, Lawson et al. (2015) reviewed the empirical evidence for the widely theorised relationship between population growth rate and environmental variability that considers the curvature of the reaction norm (Jensen's inequality) and the range over which the variation occurs. Among 17 empirical studies reviewed, almost as many studies found a positive correlation (*n* = 4) as found a negative one (*n* = 5), while five studies found evidence for a mixture of effects and three studies failed to detect an effect. Vázquez et al. (2017) reviewed the evidence for impacts of climate variability, including extreme environments, on individuals, populations, and communities, but stopped short of considering macroecological phenomena. Terry et al. (2022) developed theoretical arguments to examine how competition in temporally variable environments might affect range shifts via phenomena like storage effects and non‐linear averaging (Adler and Drake 2008; Bernhardt et al. 2018; Warner and Chesson 1985), with a continued emphasis on the curvature of reaction norms and the effects of environmental variability on fitness. Although Terry et al. (2022) gestures towards biogeographical phenomena and explicitly calls for better understanding of the effects of environmental variability at large scales, none of these studies addressed the macroecological effects of environmental variability on species distributions.

In a broad sense, ecologists have long recognised that temporal variability in conditions shapes the evolution of physiological characteristics. Janzen (1967) pointed out that lower temperature variability in the tropics compared to temperate regions leads to more limited physiological tolerances for a species and thus limits range size. Despite complications associated with elevational range and the timescale of temperature variation, Ghalambor et al. (2006) find support for most of the assertions of Janzen's (1967) climatic variability hypothesis. Extending these terrestrial insights to the ocean, planktonic duration has been shown to increase with latitude, reinforcing temperature as the primary driver of dispersal potential (Álvarez‐Noriega et al. 2020). Brown (2014) suggested that planktonically reproducing benthic marine species—effectively focused on shallow coastal or intertidal biota—would be more spatially isolated, with narrower physiological range, in the tropics compared with higher‐latitude environments. Nevertheless, Brown (2014) also recognised the complications of life history variation and how larval development modes (e.g., planktonic or not, feeding or non‐feeding) themselves exhibit latitudinal and seasonal patterns, but concluded that overall bioclimatic variation will be important for diversity and plasticity across both terrestrial and marine environments. The interaction goes both ways, as higher species diversity also mediates how ecosystems respond to environmental fluctuations (Bruno et al. 2003; Oliveira et al. 2022). Yet, the overall question of how patterns of environmental variability—themselves changing with anthropogenic climate change—will drive the evolved species' responses within (and sometimes across) biomes remains of great importance.

There are, of course, ample studies—both theoretical and empirical—that examine trends in the planetary redistribution of biodiversity in response to climate change. Some species or populations may be pre‐adapted to warmer climates than these lineages experienced throughout their evolutionary histories. Chevalier et al. (2024) introduced a new concept called “niche contiguity” to quantify this concept and provided compelling evidence for a macroecological difference between marine and terrestrial environments, with species from shallow marine environments showing greater pre‐adaptation to warming conditions than terrestrial species, and similarly greater pre‐adaptation in biodiverse equatorial environments. Comte et al. (2024) reviewed the various theories that attempt to distinguish mechanisms contributing to leading‐edge range expansion and trailing‐edge contraction as the climatological signature shifts poleward. They concluded that the relative infrequency with which functional traits are considered is hampering the ability to make progress in this area (but see Angert et al. 2011). Finally, in a comprehensive review of the theory about and evidence for climate‐induced range shifts, Lawlor et al. (2024) recommended comparing species across biomes. Here, we build upon these theories (summarised in Fig. 2 of Lawlor et al. 2024) to evaluate the role of temporal variability in macroecological patterns across biomes. Although the magnitude of temporal variation has been the primary focus of some work in population and community ecology (Baythavong 2011; Boyce et al. 2006; Lawson et al. 2015; Vázquez et al. 2017), it is typically neglected in macroecology.

Finally, our macroecological hypotheses can be motivated by thermal performance theory, which uses the concept of a thermal reaction norm to answer questions about how organisms respond to temperature fluctuations, why some species are temperature generalists while others are specialists, and why some organisms regulate their body temperature while others conform to environmental temperatures (Angilletta 2009). Importantly, much of thermal performance theory focuses on microevolutionary processes, such as genetic adaptation in unpredictable or heterogeneous environments (Kelly et al. 2012). By thinking inductively about how thermal performance might evolve for a broad collection of species, our macroecological hypotheses can be understood as the statistical generalisations of this theory. Thermal performance theory also explains acclimation, the (possibly costly) ability of individual organisms to adjust to their thermal environments through exposure (Angilletta 2009; Huey et al. 1999). To date, the literature has focused primarily on the role of mean or optimal temperatures in driving macroecological patterns and processes (Gouveia et al. 2014; Lynn et al. 2021). Here, we seek to evaluate the role of temperature variability on populations and communities at a macroecological scale.

## 1 The Variability Damping Hypothesis

Our first hypothesis—the variability damping hypothesis—posits a resistance gradient to climate‐induced stressors based on the environment's buffering capacity against temperature fluctuations. Recently, in the context of changing host–parasite relationships in marine ecosystems, it has been suggested that nearshore and intertidal ecosystems are more sensitive to climate‐induced changes than deepwater environments since water is a heat sink that will buffer deepwater populations against thermal fluctuations (Byers 2020, 2021). This is in line with our variability damping hypothesis. As an example, ‘sea star wasting’ seems exacerbated by warmer, low‐oxygen waters at the surface while sea star populations and species at greater depth show diminished effects of the disease (Dawson et al. 2023). Similarly, Ben‐Horin et al. (2013) found that the high temperature variability typical of intertidal zones increased stress and susceptibility to lethal disease in black abalone, whereas exposure to the more stable temperatures of subtidal habitats reduced infection risk. Besides temperature, of course, several climate change effects are likely important in the deep sea (Sweetman et al. 2017). We broaden the scope of this original argument to consider a variety of ecosystems that include but are not limited to, those in marine environments. For example, lake depth has a similar stabilising effect on deepwater temperature (Pilla et al. 2020). Furthermore, there is no conceptual basis for limiting these ideas to aquatic systems. Extending the continuum from marine deep water to nearshore to intertidal zones, we propose that terrestrial environments, owing to their inherently lower specific heat capacity relative to aquatic settings, are likely to undergo the most pronounced temperature fluctuations (Steele et al. 2019). Even within terrestrial environments, the effects of climate change are not uniform across the globe and some ecosystems will experience heightened variability in temperature and precipitation (Masson‐Delmotte et al. 2023). Furthermore, while earlier analysis (Byers 2020, 2021) focused on host–parasite dynamics, it's clear that the underlying principles apply to a wide array of stressors that species may encounter as a result of climate change. This holistic approach underscores the versatility and broad relevance of our argument across different ecological domains and environmental stressors.

**FIGURE 2:**
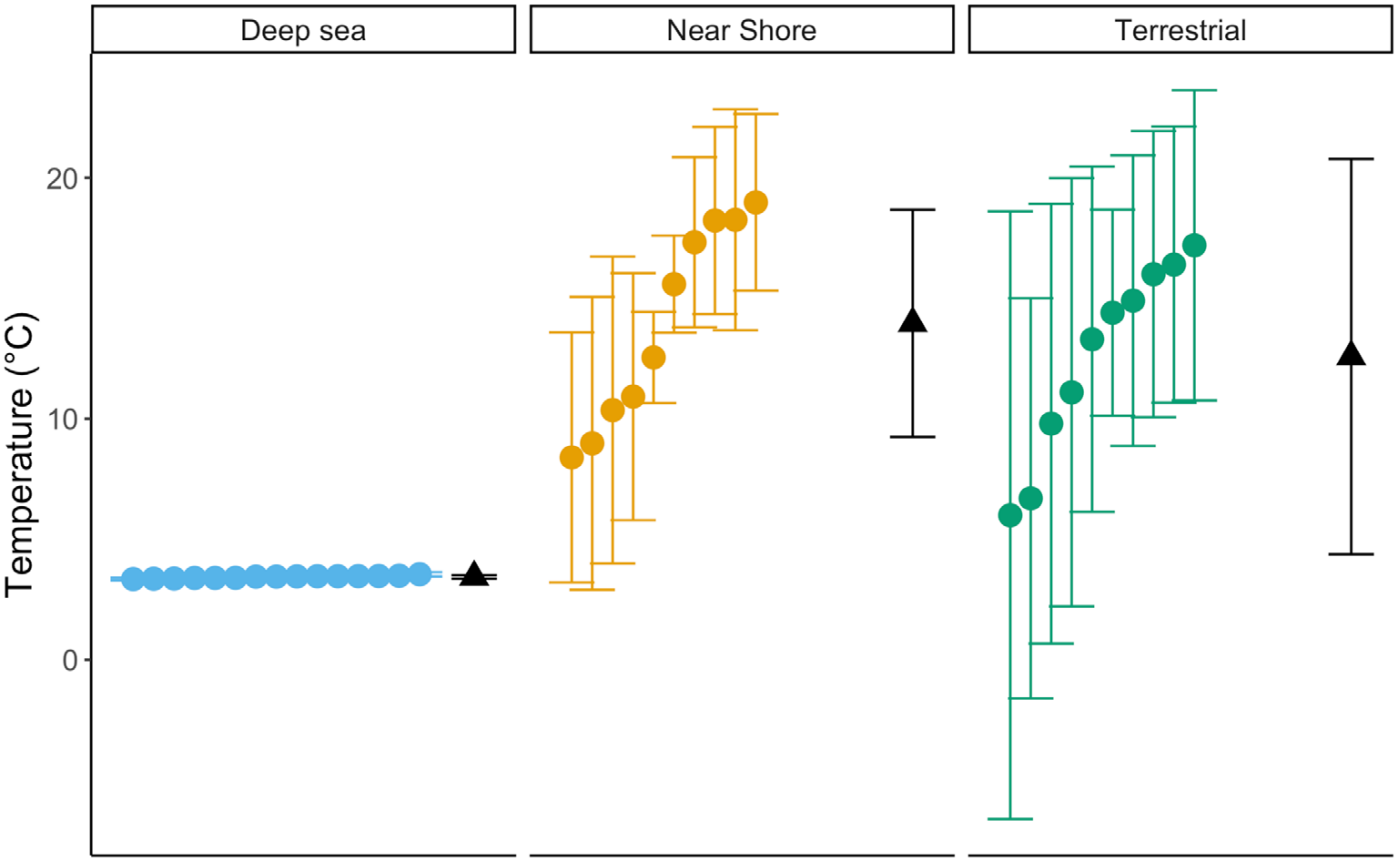
Deep sea ecosystems (*n* = 15) exhibit lower temporal variation in temperature compared with nearshore (*n* = 10) and terrestrial (*n* = 10) sites. Means and standard deviations of temperatures are presented for a 30‐year period (1991–2020), using interpolated data from the World Ocean Atlas for coastal and deep sea locations and measurements from World Meteorological Organisation weather stations for terrestrial sites. All sites were chosen at similar northern latitudes (deep sea: 42.125–42.875; coastal: 42.5; terrestrial: 42–42.985) to minimise latitudinal climate variation. Deep sea temperatures represent conditions at 2000 m depth, coastal temperatures were taken at 0 m depth, and terrestrial site elevations ranged from 4 to 396 m. Black triangles indicate the overall mean and standard deviation for each region, with deep sea locations in blue, coastal locations in light orange, and terrestrial locations in green.

We hypothesise, therefore, that resilience to fitness‐related stressors follows a specific gradient, with deep aquatic environments providing the greatest capacity to buffer physical changes (particularly heat inputs), followed by nearshore and intertidal zones, and terrestrial ecosystems being the least resistant (Figure 2). This hypothesis is grounded in the understanding that water acts as a heat sink, moderating temperature changes and thereby potentially reducing the impact of climate change on aquatic organisms compared to those on land. A slightly different version of this hypothesis considers the unique conditions of intertidal zones, which experience extreme fluctuations naturally, suggesting a revised order of resistance to change: deep water > nearshore > terrestrial > intertidal. As with all macroecological hypotheses, these ideas are meant to be a broad generalisation that will not be true in every case but may occur sufficiently widely to affect large‐scale changes in biogeographic patterns. We highlight that this hypothesis does not seek to describe major biogeographic patterns of species diversity, such as the latitudinal gradient in species richness (Fine 2015; Rohde 1992), nor is it specifically related to variation in ecosystem stability across major biomes. Instead, our hypothesis posits that environments that have greater heat absorbing capabilities will experience lower rates of species loss than those that are less buffered from increasing temperature variability and novel precipitation regimes.

## 2 The Variability Adaptation Hypothesis

What the variability damping hypothesis leaves out is the fact that species—and even populations within species—are often adapted to the conditions in which they are found (Kelly et al. 2012). Furthermore, climate change has already disrupted local adaptation in some systems, with accessions from historically warmer and drier source locations outperforming local accessions in contemporary climates (Anderson and Wadgymar 2020; Kooyers et al. 2019; Wilczek et al. 2014). Thus, it is important to consider adaptation in the light of both current and recent historical climates, along with the evolutionary histories of species (Cavender‐Bares et al. 2016). That is, the associations of species with historical fluctuation regimes may be relevant to their ability to persist through greater environmental fluctuations in the future (Jackson 1974). As an example, in both terrestrial and marine environments, distributions often shift in elevation or depth instead of latitude in response to novel climates (Chan et al. 2024); many marine species track their climatic niche through changes in depth (Poloczanska et al. 2016). High‐elevation terrestrial ecosystems typically experience heightened variability in climatic conditions relative to lower‐elevation locations, which can favour the evolution of broader climatic tolerances in high‐elevation species. Thus, it is not surprising that the magnitude and direction of range shifts induced by climate change differ substantially across elevational gradients (Mamantov et al. 2021). Similarly, the magnitude of plasticity can increase with latitude for broadly distributed species, as species in tropical ecosystems tend to experience reduced climatic variability compared with those at more poleward latitudes (Freeman et al. 2022; Louthan et al. 2021; Nati et al. 2021; Villeneuve et al. 2021). These climatic gradients could increase the susceptibility of low‐elevation and equatorial species and populations to decline, yet evaluating the vulnerability of species, populations, and communities to climate change at the global scale remains challenging (Amano et al. 2020; Louthan et al. 2021). Studies in this domain must simultaneously consider differential rates of climate change at different places on the planet, deviations from historical conditions, and variations in the breadth and optima of climatic tolerance curves (Deutsch et al. 2008). Empirical studies contrasting phylogenetically related species at different latitudes/elevations hold great promise for resolving these questions, as do global‐scale coordinated macroecological studies. One prominent example is Nati et al. (2021), who examined over 200 fish species and found that freshwater tropical species had lower variation in thermal tolerance than temperate species, implying the tropical species' greater vulnerability to thermal stress.

Building on these ideas, the second hypothesis—the variability adaptation hypothesis—challenges the variability damping hypothesis by incorporating the concept of historical adaptation to environmental fluctuations (Chevin et al. 2010; Gilchrist 1995; Lynch and Gabriel 1987; Sheldon et al. 2018). It argues that organisms that are adapted to rapid changes and variability in their environments (Baythavong 2011; Louthan et al. 2021) may actually possess a higher resilience to the stressors induced by climate change. This hypothesis proposes an inverse order of resilience: terrestrial > nearshore/intertidal > deep water. According to this idea, the preadaptation of (extratropical or high elevation) terrestrial or intertidal organisms to cyclic and seasonal change of environmental conditions could confer evolutionary protection against the novel stressors brought about by climate change. This idea emphasises the importance of evolutionary history and adaptation in shaping the resilience of species to environmental changes, such that the impact of climate change on different ecosystems cannot be predicted solely from current conditions but must also consider the historical context of species' adaptations. Consistent with this hypothesis, climate change has shifted the geographic distributions of marine species to a greater extent than terrestrial species (Lenoir et al. 2020), and species from terrestrial tropical montane ecosystems more than those inhabiting temperate mountains (Freeman et al. 2021). Taken together, these macroecological patterns reveal that species that evolved in environments with more stable climates (marine vs. terrestrial, and tropical vs. temperate) may need to alter their ranges to remain within their climatic niches, whereas species from regions of greater climatic variability may have a greater ability to persist within their contemporary ranges.

The variability adaptation hypothesis is supported by recent research on stress priming, which recognises that an organism's prior exposure to certain harsh environmental conditions can improve its survival or performance if exposed again to similar conditions (Han et al. 2025; Hilker and Schmülling 2019). Through maternal effects, epigenetic changes, and selection, stress priming can improve tolerance not only in the current generation, but also in subsequent generations (Liu et al. 2022). Thus, like the variability adaptation hypothesis, stress priming focuses on how exposure to extreme conditions makes organisms more tolerant of fluctuating conditions.

A situation that supports the variability adaptation hypothesis is ocean acidification. In the marine environment, small planktonic species with calcareous body parts that live offshore (e.g., coccolithophores and larval molluscs) are the most susceptible to acidity damage compared to their nearshore or estuarine counterparts (Boulais et al. 2017; D'Amario et al. 2020); but see (Schaum et al. 2012) for an example where the effect of variation in genotype is much larger than plasticity and adaptation. The explanation has been that offshore pH is very stable, so organisms there are adapted to very specific pH levels, and thus sensitive to even small shifts in pH. In contrast, species adapted to inshore or intertidal ecosystems are likely to have adapted to large fluctuations in pH during the daily tidal cycle (Bracken et al. 2018).

This intolerance to variation by some species has long been recognised and is encoded in terms like “stenothermal” and “stenohaline,” which describe species that tolerate a narrow range of temperature and salinity, respectively (Moore 1940). Such species often live in the open ocean where such variables are more constant. In contrast, eurythermal and euryhaline species tolerate large swings in temperature and salinity and are most often associated with estuarine environments. In estuaries, temperature and salinity typically fluctuate sizably on the scale of hours with tidal cycles. The variability adaptation hypothesis argues that eurythermal/euryhaline species tolerate climate change better than stenothermal/stenohaline species because they are adapted to extensive temporal variation in environmental conditions. Alternatively, the eurythermal and euryhaline habitats may be the most influenced by climate change because the already large swings in variables will be exacerbated, perhaps past the point of tolerance of the organisms living there (Somero 2010).

These ideas are related to how thermal performance curves may evolve in response to a directional change in temperature. Following Lynch and Lande (1993), Huey and Kingsolver (1993) developed a model for the evolution of optimal thermal performance as a quantitative trait in an infinite, sexually reproducing population subject to stabilising selection. A key innovation of this model is the characterisation of a critical rate of environmental change beyond which the population cannot persist. More relevant to our argument is the interesting finding that populations with an intermediate performance breadth are most able to adapt to a directionally changing environment (Huey and Kingsolver 1993). The intuitive explanation for this is that the fitness consequences faced by suboptimal phenotypes in populations with large performance breadth are relatively small, so that the strength of stabilising selection is too weak to exert sufficient selective pressure. On the other hand, populations with narrow performance breadth lack the variation needed for selection to be effective. Thus, the evolving optimal temperature in populations with either too large or too small a performance breadth may lag behind the changing environmental temperature too much to keep pace. From this, it is readily seen that thermal generalism is not necessarily an adaptational benefit (Angilletta 2009).

The thermal tolerance of natural populations depends not only on within‐population genetic variation, but also on the extent of gene flow across populations and the degree of local adaptation to temperature and other climatic parameters (i.e., migration‐selection balance). Traditionally, it was thought that gene flow across populations inhabiting disparate environments would constrain local adaptation to variable conditions (Slatkin 1987). However, researchers have recently recognised that asymmetrical gene flow from populations inhabiting historically warm climates into populations inhabiting historically cooler climates could stabilise those poleward or higher elevation populations under climate change (Aitken and Whitlock 2013; Bontrager and Angert 2019). That is, the classic paradigm in which gene flow restricts local adaptation may not apply in a non‐equilibrial, rapidly changing environment if gene flow introduces alleles adapted to projected climates (e.g., hotter or drier conditions) into populations that evolved under preindustrial climates. Much attention has focused on the high risk of extinction of rare species (Enquist et al. 2019). However, common species with a high degree of local adaptation to climatic factors that are changing rapidly often harbour sufficient genetic variation in thermal performance curves across multiple populations, but maintain limited genetic variation within local populations, and programmes such as assisted migration may be necessary for population persistence (Aitken and Whitlock 2013; Anderson et al. n.d.).

**FIGURE 3:**
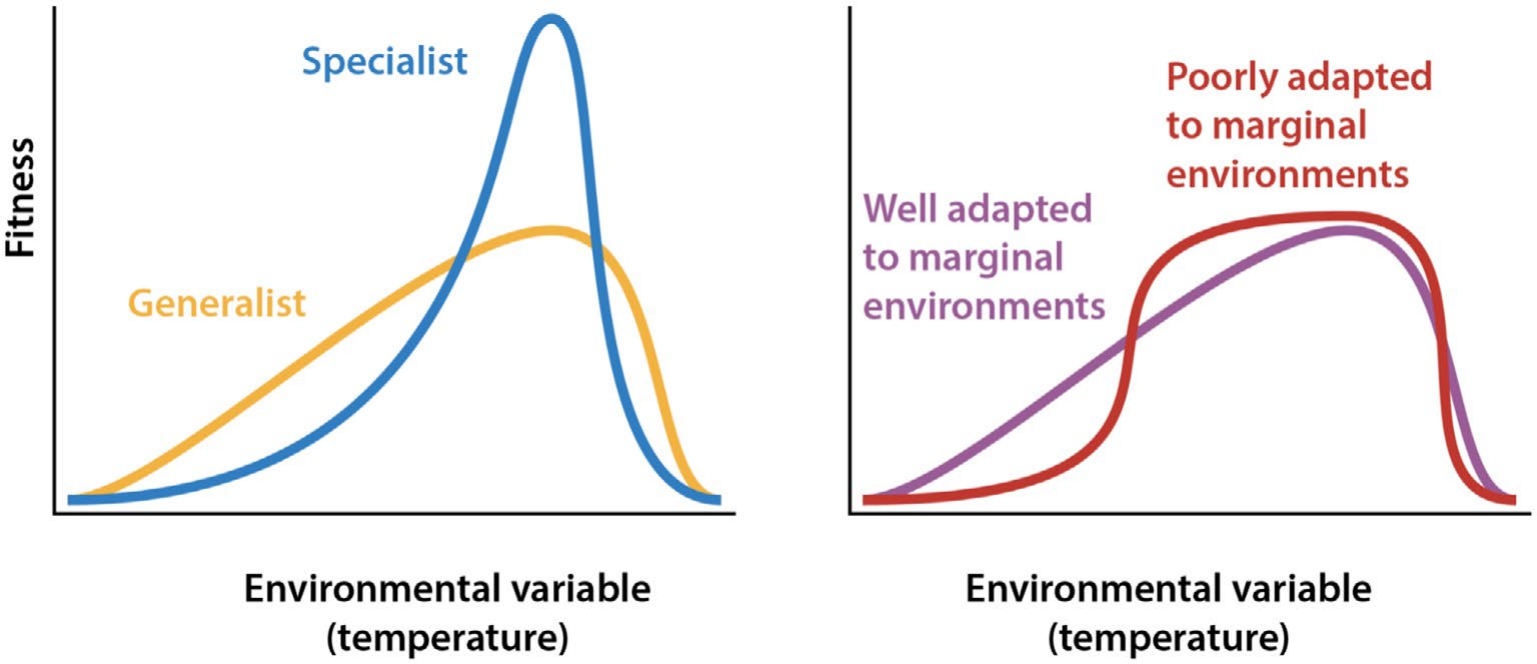
The concept of a generalist‐specialist tradeoff is well established in life history theory and concerns the relationship between peak fitness and the width of the reaction norm (i.e., tolerance window; left) (Huey and Hertz 1984; Huey and Kingsolver 1989). Here we propose that in the vicinity of their tolerances, species may be more or less adapted to fluctuations, which would be reflected in the local slope of their reaction norms near the thermal limits (right). Angilletta (2006) provides guidance on model fitting and selection for thermal performance curves.

To interrogate the variability adaptation hypothesis, one can examine how climatic variation influences the fitness of individuals of a diversity of species across ecosystems. Specifically, we suggest that the shape of the tolerance curve at the margins of a species environmental range should reflect past adaptation to environmental fluctuations and the ability to persist through phenotypic plasticity, underlying genetic variation in the population, or other mechanisms as the conditions around it change. A population adapted to climatic fluctuations is hypothesized to show a flatter tolerance curve with higher fitness under environmental extremes relative to a specialised population adapted to a specific mean climate (Figure 3). The point is not simply whether the tolerance range of a genotype is wide or narrow—the usual jack‐of‐all‐trades or master‐of‐none (Foray et al. 2014). Rather, investigating the potential for various species to adapt to climate change requires empirically measuring the shape of tolerance curves under extreme climates, especially those that reflect climatic projections. Thermal performance curves are often asymmetric (Deutsch et al. 2008; Gehman et al. 2018; Louthan et al. 2021), and increasing temperatures can rapidly depress the fitness of populations that are currently experiencing temperatures close to their optima. Furthermore, thermal performance curves measured at only one life stage can underestimate the effects of climate change, highlighting the need to model fitness components measured throughout the lifespan (Johnson et al. 2023). The fitness effects of climate change are expected to depend, therefore, not only on the speed and extent of increasing temperatures, but also on the shape of thermal performance curves, how close the current climate is to the optimal climate, and how often the temperature exceeds the critical thermal maximum temperature for a given species. We highlight that studies need to be designed to consider multiple climatic factors together (Marshall et al. 2021; Wu et al. 2024) and to examine ontogenetic shifts in climatic tolerances (Johnson et al. 2023; Sinclair et al. 2016).

## 3 Testing These Hypotheses

Returning to the macroecological consequences of climate change, we ask how the variability damping and variability adaptation hypotheses could be tested. We recognise that the hypotheses are not mutually exclusive in the sense that one or the other may hold for certain places and species; both hypotheses may have explanatory power. The challenge, of course, is that the predictions of these hypotheses are generic and concern large‐scale distribution patterns predicted to hold across species. Because there are no species with populations separately adapted to each of these major environments, there are no ways to control for species identity, yet phylogenetic relationships of species could be incorporated into statistical models to account for similar evolutionary histories. Are the hypotheses then even comparable? We suggest that there are at least two viable approaches. Both require selecting appropriate measures of resistance or resilience.

In the first case, resistance concerns changes in community composition (or lack thereof), which can be measured via species turnover or temporal beta‐diversity (Legendre 2019), local extirpation rates, similarity indices, or other community ecology metrics. Since all communities turn over at some background rate, it will be crucial to compare relative turnovers, that is, the rate of species turnover under recent contemporary climate changes compared to measurements of turnover in the past. Historical species composition could be inferred from museum or herbarium collections, surveyor notes, dissertations, and publications, or other historical records (Beller et al. 2017). Furthermore, distributed longitudinal studies could be conducted in multiple locations across time to evaluate species turnover as climate change progresses. Such studies could be embedded into the syllabi of undergraduate or graduate field courses, as long as there is continuity in data collection protocols. We highlight that any study of species turnover requires expertise in natural history and taxonomy. The variability damping hypothesis holds that relative turnover should be highest in terrestrial environments, compared with nearshore and inter‐tidal environments, and lowest in deep water environments (Astudillo‐Clavijo et al. 2024). In contrast, the variability adaptation hypothesis suggests that under increasing temporal variation in climates, species diversity would decline more in historically stable ecosystems relative to historically variable ecosystems, such that relative turnover in species is higher in deep water than nearshore and intertidal areas than tropical terrestrial habitats than extratropical environments.

The second approach to testing these hypotheses is to identify a species or two sister species (or genera or families) that live in more than one of these environments (e.g., nearshore vs. intertidal, intertidal vs. deep water) and experimentally compare relevant reaction norms in individuals from each population. Macroevolutionary approaches can link species‐level variation in traits with elements of the environment while controlling for phylogenetic relationships (e.g., Worthy et al. 2024), but quantifying the thermal breadth of numerous species is a laborious task. A useful variation of this approach would be to look within a single habitat that has extreme physical gradients, such as an estuary. The middle of an estuary often has the highest variation in most physical properties, including temperature, because it is the intersection between freshwater inputs from upstream and marine inputs from the ocean, and the predominance of these inputs varies with the tidal cycle. Thus a single species living at various positions along this gradient would experience very different variation in environments. Of course, model species for this approach may be difficult to identify. Moreover, the approach depends on selecting species whose life histories already allow them to live in multiple environments (i.e., species that have already evolved as generalists). In one respect, this approach is the more direct: it controls for many aspects of species identity that could otherwise be confounding. In other respects, it is less direct, as (1) it does not consider the standing genetic variation affecting the measured reaction norms (although if one were to sample enough accessions from within a population, this standing genetic variation could be accounted for by modelling performance curves of multiple genotypes), and (2) it only addresses the underlying presumed genetic and ecological capacity for resilience, not resilience itself.

Based on the hypotheses we have outlined, we believe these concepts are empirically testable and are distinctly different from one another. Furthermore, our reflections indicate that while testing these hypotheses presents complex challenges, it remains a viable and potentially rewarding avenue for future research. This suggests that pursuing these lines of inquiry could yield significant insights into the mechanisms driving species' responses to environmental change.

## Author Contributions

J.M.D. initiated the research reported here and wrote the first draft. All authors participated in developing the ideas and contributed substantially to the manuscript.

## Data Availability

Data in Figure 2 were obtained from the World Ocean Atlas (https://www.ncei.noaa.gov/access/world‐ocean‐atlas‐2023/) for marine sites and the World Meteorological Organisation (https://www.ncei.noaa.gov/data/oceans/archive/arc0216/0253808/5.5/data/0‐data/data‐composite‐primary‐parameters‐min24/) for terrestrial sites. Code and data to generate Figure 2 is available online at DOI: 10.5061/dryad.1zcrjdg42.
